# SARS-CoV-2 Antibody Seroprevalence in Gabon: Findings from a Nationwide Household Serosurvey in a Sub-Saharan Africa Country

**DOI:** 10.3390/v16101582

**Published:** 2024-10-09

**Authors:** Samira Zoa-Assoumou, Paulin Essone-Ndong, Rafiou Adamou, Sandrine Lydie Oyegue-Liabagui, Amandine Mveang Nzoghe, Bayodé Roméo Adegbite, Armel Mintsa Ndong, Herve Mboyis-Kandem, Marien Juliet Verraldy Magossou Mbadinga, Angelique Ndjoyi-Mbiguino, Armel Brice Amalet Akagha, Krystina Mengue Me Ngou-Milama, Magaran Monzon Bagayoko, Inoua Aboubacar, Jean-Bernard Lekana-Douki, Joel Fleury Djoba Siawaya, Ayola Akim Adegnika, Edgard-Brice Ngoungou, COVID-19 Gabonese Group

**Affiliations:** 1 Département de Bactériologie-Virologie, Université des Sciences de la Santé (USS), Libreville BP 4009, Gabon; hervemboyis5@gmail.com (H.M.-K.); labmicus@gmail.com (A.N.-M.); 2 Laboratoire Professeur Daniel Gahouma (LPDG), Libreville BP 4009, Gabon; 3 Centre de Recherches Médicales de Lambaréné (CERMEL), Lambaréné BP 118, Gabon; paulinessone@gmail.com (P.E.-N.); adamourafiou@gmail.com (R.A.); aromakobs@yahoo.fr (B.R.A.); ayola-akim.adegnika@uni-tuebingen.de (A.A.A.); 4 Laboratoire Nationale de Santé Publique (LNSP), Libreville BP 4009, Gabon; a_mintsa@yahoo.fr; 5 Unité d’Evolution, Epidémiologie et Résistance Parasitaire, Centre Interdisciplinaire de Recherches Médicales de Franceville (CIRMF), Franceville BP 769, Gabon; lyds_ass@yahoo.fr (S.L.O.-L.); lekana_jb@yahoo.fr (J.-B.L.-D.); 6 Département de Biologie, Faculté des Sciences, Université des Sciences et Techniques de Masuku (USTM), Franceville BP 942, Gabon; 7 Unité de Recherche et Diagnostics Spécialisé, Service Laboratoire, CHU-Mère-Enfant Fondation Jeanne Ebori, Libreville BP 4009, Gabon; nzogheamandine@yahoo.fr (A.M.N.); julietveraldy@yahoo.fr (M.J.V.M.M.); joel.djoba@gmail.com (J.F.D.S.); 8 Organisation Mondiale de la Santé, Libreville BP 4009, Gabon; amaletb@who.int (A.B.A.A.); ngoukrystina@yahoo.fr (K.M.M.N.-M.); bagayokom@who.int (M.M.B.); inouaa@who.int (I.A.); 9 Département de Parasitologie-Mycologie Médecine Tropicale, Faculté de Médecine, Université des Sciences de la Santé (USS), Libreville BP 4009, Gabon; 10 Institut für Tropenmedizin, Universität Tübingen, German Center for Infection Research (DZIF), 72074 Tübingen, Germany; 11 Department of Parasitology, Leiden University Medical Center, 2333 ZA Leiden, The Netherlands; 12 Department of Epidemiology-Biostatistics and Medical Informatics (DEBIM), Faculty of Medicine, University of Health Sciences, Libreville BP 4009, Gabon

**Keywords:** population-based survey, antibody, COVID-19, SARS-CoV-2, seroprevalence, Gabon

## Abstract

Seroconversion surveys of anti-SARS-CoV-2 antibodies provide accurate estimates of the prevalence of SARS-CoV-2 infections. This nationwide population-based cross-sectional serosurvey aimed to evaluate the prevalence of SARS-CoV-2 antibodies among residents in Gabon and compare the estimated cumulative number of COVID-19 cases with the officially registered number of laboratory-confirmed cases up to December 2021. Households in each province were randomly selected. Twenty-eight localities, including sixteen urban and twelve rural, were randomly selected for the study. Whole blood samples were collected in dry tubes from all study participants nationwide within 15 days. Serum samples were used to measure SARS-CoV-2-specific ELISA titers. Overall, data from 1672 households were analyzed. Out of the 3659 participants, 3175 were found to be positive for SARS-CoV-2 antibodies, resulting in a crude seroprevalence of 86.77%. Stratification of study participants by age group showed the highest seroprevalences in the 20–29 and 40–49 age groups with 91.78% (95% CI: 89.5–93.6) and 91.42% (95% CI: 88.7–93.5), respectively. Nyanga province had the lowest prevalence (72.8%), and Estuaire and Ogooué-Lolo provinces had the highest prevalence (90 and 92%). Our results suggest a high transmission rate in the Gabonese population 21 months after the first SARS-CoV-2 case in the country. This high seroprevalence estimate could indicate that the population may not have adequately implemented or appropriately adhered to the applied infection control measures.

## 1. Introduction

The coronavirus disease 2019 (COVID-19) has spread and caused high morbidity and mortality worldwide since it was first reported in Wuhan in the Chinese province of Hubei in late 2019 [1,2]. In Central Africa, Gabon was the third most affected country by COVID-19 behind Cameroon and the Democratic Republic of Congo, with 48,980 cases and 306 deaths of critically ill patients since the first confirmed case of COVID-19 on 12 March 2020 (https://africacdc.org/COVID-19/, accessed on 12 December 2023). 

The response strategy against COVID-19 in Gabon was based on tests performed on travelers, suspected cases, and direct contacts of people who tested positive in the country and the implementation of prevention and control measures [3]. 

In this country of 2.1 million inhabitants, a network of 15 RT-PCR laboratories located across the country with 60 sampling sites performed between 2000 and 5000 tests per day.

Moreover, in a previous cross-sectional study conducted in Libreville, Gabon, the mortality rate associated with COVID-19 infection from March to June 2020 was shown to be low, and patients who died of COVID-19 infection were found to be younger on average than reported elsewhere [4].

Between 12 March 2020 and 20 November 2021, the country tested 266,271 people and recorded a total of 9069 SARS-CoV-2 RT-PCR-positive cases (3.4% infection rate), 8938 patients who were cured (98.5% cured rate), 65 active cases, and 58 deaths (0.6% case fatality rate) (Country report).

Although massive screening campaigns were occasionally organized in the country, the actual infection rate of severe acute respiratory syndrome coronavirus 2 (SARS-CoV-2), the causative agent of COVID-19, remains unclear since asymptomatic individuals may represent the hidden face of the iceberg and, therefore, a major source of viral spread. Case reporting is influenced by strategies implemented for case finding, testing, and contact tracing and may underestimate the burden of SARS-CoV-2 infection. 

As recommended by the World Health Organization (WHO), measuring the extent of seropositivity could estimate the proportion of individuals positive for SARS-CoV-2 antibodies in the population. It could further indicate the rate of disease transmission over time [5]. Moreover, as the extent of infection is heavily related to social interactions, population density and the assessment of the proportion of potentially protected individuals in the population help the authorities to plan public health interventions. Population-based seroepidemiological studies help measure the extent of SARS-CoV-2 infection and the effect of ongoing public health responses on controlling the pandemic [5]. Due to the increased number of hospital cases and patients, restrictions on mass gatherings were initiated in April 2020. These restrictions were accompanied by a vaccination campaign as a measure to control the pandemic. Indeed, Gabon began its vaccination campaign on 23 March 2021, and it is based on the Sinopharm vaccine. As of 18 September 2021, 100,884 people had been fully vaccinated with a first dose out of a target population of 1,138,000 who are eligible for vaccination, or 9.93%, with more men having been vaccinated (79%) compared to women (21%) [6].

This study aimed to conduct a national household serosurvey to estimate the nationwide seroprevalence of SARS-CoV-2 antibodies in the general population, including age group, sex, area of residence, and COVID-19-related characteristics, and to determine trends in infection since the implementation of restrictive measures and vaccination campaigns.

## 2. Materials and Methods

### 2.1. Study Design and Population

This study was designed by WHO united studies [7]. This population-based cross-sectional study was conducted across the entire Gabonese Territory (the main cities of all provinces and villages in rural areas drawn by lot) between 28 November and 20 December 2021.

Gabon is a Central African country with an area of approximately 268,000 km^2^, located on the edge of the Atlantic Ocean and bordering Congo-Brazzaville, Cameroon, and mainland Equatorial Guinea. Its population, one of the smallest in Central Africa, is estimated at 1,811,079 inhabitants. Gabon is subdivided into nine provinces, with a capital in each province and 52 departments.

### 2.2. Sample Collection

We randomly selected and invited participants from the general population and areas included in the study according to specific inclusion and exclusion criteria. Individuals of both sexes aged one year and over and residing for more than three months in the study area were eligible to participate in the study. Individuals not living in the locality surveyed, those residing for less than three months, and those who refused to provide informed consent and contraindications for venipuncture were excluded.

Random selection was performed at two levels. The first category concerned the areas to be surveyed. This random selection was made from the national census file of 2013 households distributed in the enumeration areas. Subsequently, in each of the areas selected in the first stage, a sampling interval was calculated with a fixed number of 20 households to be visited. The survey teams randomly chose a starting household and visited it at an established sampling interval. All eligible permanent-resident household members present at the time of the survey team visits were invited to participate. At least one individual from each random household was enrolled in a serological survey.

Before their enrollment in the study, written informed consent from individuals aged 18 years or older or assent from children aged between 12 and 17 years, with written informed consent from their parents or guardians, was obtained.

The study was conducted using the Declaration of Helsinki, ICH-GCP guidelines, and local regulations. The protocol was approved by the National Ethics Committee for Research (0056/2021/P/SG/CNER).

### 2.3. Procedures

Eligible participants were interviewed to collect information about their sociodemographic characteristics, medical history, recent COVID-19-related symptoms (e.g., fever, cough, fatigue, loss of taste or smell, sore throat), and COVID-19-related exposure and SARS-CoV-2 vaccination using the KOBOCOLLECT mobile phone application (kc.kobotoolbox.org) on a questionnaire that included GPS data. Paper questionnaires were also made available to the teams to address difficulties in the operation of tablets and telephones, particularly in rural areas. After the questionnaire was completed, a research nurse collected 5 mL of venous blood in dry tubes. WHO-recommended WANTAI Elisa Kits were used according to the manufacturer’s instructions to assess the presence of SARS-CoV-2 spike protein-specific antibodies in serum samples. This assay has a sensitivity of 94.36% and a specificity of 100% (https://www.ystwt.cn/wp-content/uploads/2020/05/Brochure-Wantai-SARS-CoV-2-Ab-ELISA.pdf, accessed on 10 October 2021). The assay was calibrated with positive and negative quality controls before sample analyses. Assay results higher than or equal to the cutoff index value were interpreted as positive for SARS-CoV-2 antibodies. As part of the quality control, 5% of the 3455 serum samples were re-tested using the same assay by two different laboratory technicians.

### 2.4. Concordance Test

The manufacturer-reported sensitivity and specificity of the ELISA kits (WANTAI) were 96.7% (95% confidence interval [CI], 83.3–99.4) and 97.5% (95% CI, 91.3–99.3), respectively. These values were based on 30 SARS-CoV-2 antibody-positive serum samples confirmed by a nucleic acid amplification test (NAAT) for both IgM and IgG antibodies and 80 antibody-negative serum samples collected before the SARS-CoV-2 pandemic.

An independent concordance test was performed between WANTAI Elisa and Elecsys^®^Anti-SARS-CoV-2 using 100 positive and 85 negative randomly selected serum samples tested using the WANTAI Elisa kits. All of the 100 samples positive for WANTAI Elisa were positive for Elecsys^®^Anti-SARS-CoV-2, corresponding to a collective sensitivity of 100%; 77 of the 85 negative samples tested negative for both of the ELISA kits used, corresponding to a collective specificity of 90.6%. 

### 2.5. Sample Size Estimation and Statistical Analyses

The sample size needed to estimate prevalence in the study was calculated to be 3455 based on a seroprevalence rate of 10%, with a confidence level of 95%, precision of 2%, and calculation factor of 4 (https://www.openepi.com/SampleSize/SSPropor.htm, accessed on 6 September 2021). The required sample size for each area was proportional to each area’s population relative to the country’s total population.

Demographic variables included age, sex, area of residence, and school level. COVID-19-related symptoms included cough, fever, sore throat, headache, diarrhea, altered level of consciousness, and chest pain experienced during the 12 weeks preceding questionnaire completion. Moreover, data on vaccination status were recorded.

The dependent variable was the presence or absence of anti-SARS-CoV-2 antibodies, and the other collected variables were the independent variables. 

Data were analyzed using the R statistical software (The R Foundation for Statistical Computing, Vienna, Austria). Qualitative variables were described using percentages, and quantitative variables using mean ± SD, and median (min and max). A 95% confidence interval (CI) was calculated for all the prevalence values. The significance level was set at 5%.

## 3. Results

### 3.1. Study Profile

**Figure 1:**
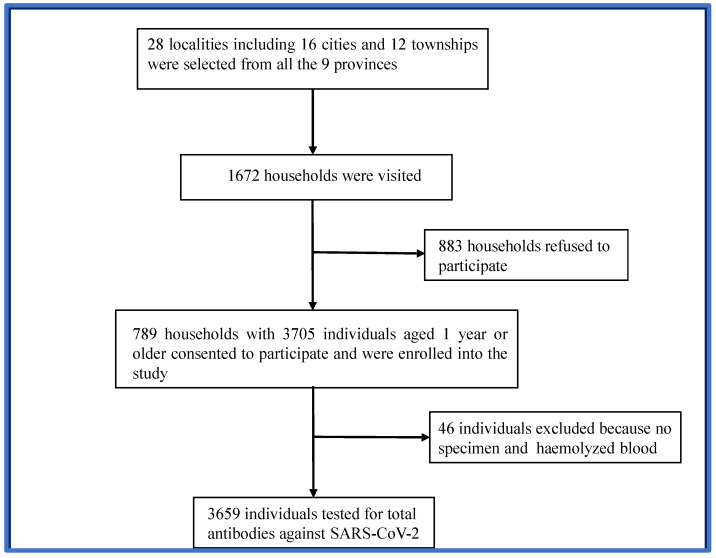
Flowchart of participant enrollment.

Based on the size of the Gabonese population in the nine provinces, 28 localities, including 16 urban and 12 rural areas, were randomly selected to participate in the study. In total, 1672 households were visited during the study period. Approximately 47.2% (789) of the households consented to participating in the study. Of these, 3705 eligible individuals aged 1 year and above were enrolled (Figure 1). Forty-six (46) individuals were excluded from the analysis because of the poor quality of the samples provided. Therefore, 3659 participants were examined for the presence of SARS-CoV-2 antibodies.

### 3.2. Demographic Characteristics of Study Participants

The participants’ main characteristics are shown in Table 1. An analysis of the study population showed that the 30–39 age group (20.6%) was the most represented, followed by the 20–29 (19.5%), 40–49 (15.2%), and 50–59 (11.2%) age groups. Children aged 0–4 (2.7%) and those aged >70 years (3.9%) were the least represented age groups. The mean age was 32.6 years (standard deviation [SD]: 23). The sex ratio was 1 (50.6 F/49.6 M). The sex profile of our baseline population was consistent with that of the entire Gabonese population. Among all participants, 14.88% resided in rural areas, while 85.46% resided in urban areas. This is consistent with the general population, in which 87% live in urban areas and 13% in rural areas.

### 3.3. Seroprevalence in the Study Population

A total of 3175 individuals tested positive for SARS-CoV-2 antibodies. Only 484 participants tested negative for SARS-CoV-2. The national seroprevalence was 86.8% (95% CI: [86–88]). No statistical difference in seroprevalence was found between women (87.82%, 95% CI: [86.2–89.2]) and men (86.45%, 95% CI: [84.8–87.9]). Stratification of the study participants by age group showed the highest seroprevalences in the 20–29 and 40–49 age groups at 91.78% (95% CI: 89.5–93.6) and 91.42% (95% CI: 88.7–93.5), respectively (Table 2).

The prevalence of SARS-CoV-2 antibodies was similar among the age groups [10, 14], [15, 19], and [30, 39] at 87.6% (95% CI: 83–91), 87.5% (95% CI: 83–91), and 88.3% (95% CI: 84–91), respectively. The seroprevalence was higher in the most active study groups from 10 to 49 years of age (school-age and young workers) and was found to be between 87 and 91%. This seroprevalence was reduced in participants aged 50 years and older and was found to be between 81 and 82.6%. The lowest seroprevalence was found in children, with 72.6% (95% CI: 62–91) for those aged 0–4 years and 79.6% (95% CI: 74–85) for those aged 5–9 years.

Nyanga is a less-populated province in Gabon (Table 3). Only 2% of the study population were enrolled in this province. It also presented the lowest vaccine coverage, with only 4% (three participants) of the study participants from this province being vaccinated. The provinces with the lowest seroprevalence in Gabon, Nyanga and Ngounié (72, 8, and 77.2%, respectively), were located in the south. Moyen Ogooué had the highest vaccine coverage.

In the province, only 12 of 134 participants (9%) were vaccinated. The comorbidity rate was also high in this province, with more than 15% of the participants having at least one comorbidity. Estuaire is the most populated province in Gabon, and more than 50% of the study population was recruited from this province. The vaccine coverage here was 7.4%, and 9.2% of the study participants had at least one comorbidity.

The prevalence of seropositivity varied most substantially according to the presence of COVID-19 symptoms in the previous three months, history of COVID-19 diagnosis, and immunization records (Table 4).

The four main cities in this province were selected for this study: Akanda, Owendo, Libreville, and Ntoum (Table 5). Owendo had the highest rate of comorbidities, with 19% of participants from that city presenting at least one comorbidity. The seroprevalence was also high in that city (92.8%). Akiéni is a small rural area within the Haut-Ogooué province. Eleven participants were enrolled in the study. All the patients tested positive for SARS-CoV-2 antibodies (100% seroprevalence). Only Lambaréné (Moyen-Ogooué) was the only place a seroprevalence < 70% in Gabon. The remaining cities had seroprevalence rates ranging from 84% to 100%.

## 4. Discussion

Gabon is one of the countries most affected by SARS-CoV-2 in Central Africa. However, in the absence of seroprevalence studies, the actual infection rate in Gabon remains unclear. This study aimed to estimate the seroprevalence of SARS-CoV-2 antibodies across Gabon. This study sampled largely populated and geographically distributed regions. All of the main Gabonese cities (urban) and randomly selected rural (village) areas were visited within 15 days. The National seroprevalence was estimated to be 86%, providing estimates of the proportion of SARS-CoV-2 infection among Gabonese people.

This is the first study to reveal a high SARS-CoV-2 seroprevalence with shallow vaccine coverage at the population or country level (https://www.nytimes.com/interactive/2021/world/covid-vaccinations-tracker.html, accessed on 10 July 2022). The results presented here are not surprising, as many studies have shown the rapid progression of SARS-CoV-2 antibody seroprevalence in the African population. The data in this study were collected 21 months after the first SARS-CoV-2 infection in Gabon (March 2020 to November 2021). The seroprevalence observed in our study might be explained by the modes of transmission of this virus (airborne) and by the low compliance of the general population with applied health regulations such as physical distancing and wearing masks [8].

Sagara et al. collected blood samples from one urban and two rural communities in Mali during two separate visits within six months [9]. Within this period, the seroprevalence was multiplied by more than three in urban areas, from 19% during the first visit (July to October 2020) to 70% during the second visit (December 2020 to January 2021). A tenfold increase was observed in the rural areas during the same period. This corroborates the observations made in South Africa, where the authors collected blood from urban and rural cohorts every two months from July 2020 to March 2021 [10]. During this nine-month study, the seroprevalence increased from 1% to 26% in rural settings and from 15% to 41% in urban communities after the two waves of COVID-19. A hospital-based study enrolling patients and parents visiting a health center in Libreville (Gabon) revealed a seroprevalence of 36% six months after the first detection of SARS-CoV-2 in Gabon [11]. Although morbidity and mortality were low in Central Africa, this region was found to have the highest seroprevalence in a systematic review aimed at estimating global and regional SARS-CoV-2 seroprevalence worldwide [12]. Possible explanations for the low mortality in Africa include the demographic pyramid, trained immunity, genetics, and broader sociocultural dynamics [13,14,15]. Moreover, many African countries have also implemented partial or complete travel restrictions, which further reduce the introduction rate of imported cases, thus making it easier to identify and isolate initial cases and their contacts and limit pockets of transmission [16].

The data presented in the current study, showing naturally acquired immunity in the Gabonese population after 21 months of COVID-19 emergence, corroborates the results of a previous survey. The SARS-CoV-2 transmission rate in urban and rural settings is high in Central Africa, especially in Gabon. Further studies are needed to determine the source of the observed resistance to COVID-19 in the Gabonese population. Indeed, of the 1,622,064 tests carried out, only 48,981 positive cases were recorded, with 306 deaths.

The presented data showed the highest transmission rate among school-aged and young workers aged 10 to 49 years. This observation can be explained by the fact that the younger population may have adopted fewer safety measures to limit transmission. Notably, the rates of morbidity and mortality due to COVID-19 are low in this age range. Morbidity and mortality due to COVID-19 have been associated with age structure and co-endemicity with other pathogens, including helminths and other related viruses [17]. This profile was already shown in the early stages of the COVID-19 pandemic in a seroprevalence study conducted in Kenya eight months after the introduction of SARS-CoV-2 to this country [18]. The seroprevalence was found to be between 30 and 39% in the age group between 10 and 59 years old but 22% in participants older than 60 years. The lowest prevalence was observed in the younger population (<10 years old) at 19%. Some studies have shown the highest seroprevalence in older population [19,20].

## 5. Conclusions

The presented data show a high transmission rate of the first SARS-CoV-2 in Gabon. The virus was first detected in March 2020 in Gabon. After 21 months, the population presented with a high SARS-CoV-2 seroprevalence, indicating a high prevalence of asymptomatic or mildly unreported COVID-19 cases in the country.

## Figures and Tables

**Table 1:** Baseline characteristics by age group.

Age	Population Size	Sex	Mean Age, Years (SD)	Comorbid Conditions		SARS-CoV-2 Vaccination	
		Male, n (%)		Yes n (%)	No, n (%)	Yes, n (%)	No, n (%)
[1, 4]	95	45 (47.4)	3 (1)	0 (0)	95 (100)	2 (2.1)	93 (97.9)
[5, 9]	221	117 (52.9)	7 (1)	0 (0)	221(100)	1 (0.5)	220 (99.5)
[10, 14]	282	123 (43.6)	12 (1)	1 (0.4)	281 (99.6)	0 (0)	282 (100)
[15, 19]	248	117 (47.2)	17 (1)	5 (2)	243 (98)	4 (1.6)	244 (98.4)
[20, 29]	707	335 (47.4)	25 (3)	28 (4)	679 (96)	42 (5.9)	665 (94.1)
[30, 39]	746	352 (47.2)	34 (3)	32 (4.3)	714 (95.7)	69 (9.2)	677 (90.8)
[40, 49]	550	292 (53.1)	44 (3)	53 (9.6)	497 (90.4)	64 (11.6)	486 (88.4)
[50, 59]	407	227 (55.8)	54 (3)	64 (15.7)	343 (84.3)	40 (9.8)	367 (90.2)
[60, 69]	217	113 (52.1)	64 (3)	57 (26.3)	160 (73.7)	19 (8.8)	198 (91.2)
≥70	144	72 (50)	76 (6)	47 (32.6)	97 (67.4)	9 (6.2)	135 (93.8)
Overall	3659	1793 (49)	32.6 (6)	287 (7.8)	3372 (92.2)	250 (6.8)	3409 (93.2)

SD, standard deviation.

**Table 2:** Seroprevalence of SARS-CoV-2 by age group.

Age	Population Size	Seroprevalence, % (95% CI)
[1, 4]	95	72.6 (62–81)
[5, 9]	221	79.6 (74–85)
[10, 14]	282	87.6 (83–91)
[15, 19]	248	87.5 (83–91)
[20, 29]	707	91.7 (89–94)
[30, 39]	746	88.3 (86–91)
[40, 49]	550	91.1 (88–93)
[50, 59]	407	82.6 (78–86)
[60, 69]	217	81.1 (75–86)
≥70	144	82.6 (75–88)
Overall	3659	86.8 (86–88)

CI, confidence interval; SARS-CoV-2, severe acute respiratory syndrome coronavirus 2.

**Table 3:** Seroprevalence of SARS-CoV-2 by province.

Provinces	Population Size	Sex	Mean Age, Years (SD)	Comorbid Conditions		SARS-CoV-2 Vaccination		Seroprevalence,% (95% CI)
		Male, n (%)		Yes, n (%)	No, n (%)	Yes, n (%)	No, n (%)	
Estuaire	1890	967 (51.2)	36 (16)	173 (9.2)	1717 (90.8)	139 (7.4)	1751 (92.6)	89.9 (88–90)
Haut Ogooué	504	239 (47.4)	33 (21)	21 (4.2)	483 (95.8)	33 (6.5)	471 (93.5)	85.8 (83–89)
Moyen Ogooué	134	59 (44)	29 (21)	21 (15.7)	113 (84.3)	12 (9)	122 (91)	85.2 (78–91)
Ngounié	181	84 (46.4)	33 (23)	18 (9.9)	163 (90.1)	8 (4.4)	173 (95.6)	77.2 (71–83)
Nyanga	75	43 (57.3)	36 (23)	5 (6.7)	70 (93.3)	3 (4)	72 (96)	72.8 (64–81)
Ogooué Ivindo	171	93 (54.4)	27 (21)	13 (7.6)	158 (92.4)	15 (8.8)	156 (91.2)	78.4 (72–84)
Ogooué Lolo	121	61 (50.4)	35 (21)	15 (12.4)	106 (87.6)	9 (7.4)	112 (92.6)	92.7 (87–96)
Ogooué Maritime	272	128 (47.1)	38 (16)	13 (4.8)	259 (95.2)	19 (7)	253 (93)	81.7 (77–86)
Woleu-Ntem	272	121 (44.5)	26 (17)	8 (2.9)	264 (97.1)	13 (4.8)	259 (95.2)	87.5 (83–91)

CI, confidence interval; SD, standard deviation; SARS-CoV-2, severe acute respiratory syndrome coronavirus 2.

**Table 4:** Seroprevalence of SARS-CoV-2 by history of respiratory symptoms and immunization records.

Characteristics	Population Size	Number of Positive	Seroprevalence, % (95% CI)
History of respiratory symptoms in previous three months			
yes	1896	1697	89.5 (88–91)
No	1719	1478	86.0 (84–88)
History of COVID-19 diagnosis			
yes	74	65	87.8 (79–94)
no	3116	2707	86.9 (86–88)
do not know	425	378	89.0 (86–92)
Immunization records			
yes	251	234	93.2 (90–96)
no	3364	2916	86.7 (86–88)

**Table 5:** Seroprevalence of SARS-CoV-2 by city.

Cities	Population	Sex	Mean Age, Years (SD)	Comorbid Conditions		SARS-CoV-2 Vaccination		Seroprevalence, % (95% CI)
		Male, n (%)		Yes, n (%)	No, n (%)	Yes, n (%)	No, n (%)	
Akanda	75	37 (49.3)	30 (17)	8 (10.7)	67 (89.3)	10 (13.3)	65 (86.7)	89.3 (80–95)
Akieni	11	7 (63.6)	34 (17)	0 (0)	11 (100)	1 (9.1)	10 (90.9)	100 (68–100)
Bitam	91	46 (50.5)	24 (15)	1 (1.1)	90 (98.9)	4 (4.4)	87 (95.6)	92.3 (84–97)
Franceville	220	99 (45)	31 (19)	12 (5.5)	208 (94.5)	20 (9.1)	200 (90.9)	94.5 (91–97)
Koulamoutou	59	31 (52.5)	30 (19)	6 (10.2)	53 (89.8)	9 (15.3)	50 (84.7)	96.6 (87–99)
Lambaréné	102	44 (43.1)	26 (21)	10 (9.8)	92 (90.2)	10 (9.8)	92 (90.2)	67.6 (58–76)
Libreville	1447	751 (51.9)	35 (15)	124 (8.6)	1323 (91.4)	121 (8.4)	1326 (91.6)	90 (88–92)
Makokou	118	62 (52.5)	26 (19)	8 (6.8)	110 (93.2)	15 (12.7)	103 (87.3)	84.7 (77–91)
Moanda	83	31 (37.3)	23 (15)	0 (0)	83 (100)	1 (1.2)	82 (98.8)	85.5 (76–92)
Mouila	105	47 (44.8)	26 (20)	7 (6.7)	98 (93.3)	6 (5.7)	99 (94.3)	88.6 (81–94)
Ntoum	161	85 (52.8)	39 (18)	9 (5.6)	152 (94.4)	3 (1.9)	158 (98.1)	88.2 (82–93)
Okondja	57	35 (61.4)	35 (14)	5 (8.8)	52 (91.2)	8 (14)	49 (86)	86 (74–93)
Owendo	166	73 (44)	39 (15)	32 (19.3)	134 (80.7)	3 (1.8)	163 (98.2)	92.8 (87–96)
Oyem	139	57 (41)	27 (18)	6 (4.3)	133 (95.7)	9 (6.5)	130 (93.5)	86.3 (79–91)
Port-Gentil	213	94 (44.1)	37 (15)	10 (4.7)	203 (95.3)	18 (8.5)	195 (91.5)	86 (81–91)
Tchibanga	31	13 (41.9)	26 (17)	0 (0)	31 (100)	3 (9.7)	28 (90.3)	87.1 (69–96)

CI, confidence interval, SARS-CoV-2, SD, standard deviation; severe acute respiratory syndrome coronavirus 2.

## Data Availability

A subset of the key-anonymized individual data collected during the study, along with a data dictionary, is available upon request from the corresponding author at ngoungou2001@yahoo.fr after a proposal with a signed data access agreement is approved.
